# Cyclic Cushing's Disease in the Prepubertal Period—A Case Report and Review of Literature

**DOI:** 10.3389/fendo.2019.00701

**Published:** 2019-10-18

**Authors:** Anna Wȩdrychowicz, Barbara Hull, Anna Kalicka-Kasperczyk, Grzegorz Zieliński, Jerzy B. Starzyk

**Affiliations:** ^1^Department of Pediatric and Adolescent Endocrinology, Pediatric Institute, Medical College, Jagiellonian University, Kraków, Poland; ^2^Department of Neurosurgery, Military Institute of Medicine, Warsaw, Poland

**Keywords:** cyclicity, hypercortisolemia, prepubertal age, Cushing's disease, Cushing's syndrome

## Abstract

**Background:** Cyclic Cushing's disease (CD) has been described in about 15% of adult patients with CD. In the pediatric population, diagnosis of CD is rare and cyclic presentations of the disease are not adequately understood or described. Moreover, prepubertal patients usually do not present with the typical signs and symptoms of CD, which can obscure or delay diagnosis. In this paper, we report a case of cyclic CD in a prepubertal age girls whose etiology was a pituitary corticotropinoma.

**Case presentation:** A Caucasian 7.8 year old girl was admitted to pediatric endocrinology for the evaluation of short stature and prior obesity. The patient remained overweight despite significant lifestyle modifications, resulting in 6 kg weight loss during the prior 6 months. The physical exam was notable for precocious adrenarche and thelarche, but difficult to differentiate from steatomastia. Hypothalamo-pituitary-adrenal axis diagnostics, including single diurnal excretion of urinary cortisol, morning ACTH, and serum cortisol levels, were all within normal limits, and MRI of the pituitary gland showed no deviations at this time. Because of the clinical suspicion of cyclic hypercortisolemia, she was referred to our outpatient clinic for follow-up. After 6 months, the patient returned with rapid weight gain, accompanied by nocturnal anxiety, exacerbation of depressive behavior, insomnia and excessive sweating, and was readmitted to the ward for testing. Standard diagnostics confirmed CD and repeat MRI at 8.6 years old showed a microadenoma of 3 × 4 mm in the right side of the anterior pituitary gland. Histopathologic examination described an atypical, densely-granulated pituitary corticotroph adenoma with Ki-67 expression above 3%.

**Conclusion:** Cyclic presentations of CD in the prepubescent age group could cause difficulties in diagnosis because of atypical signs and symptoms, which can be absent in the remission phase. Decreases in height percentiles and velocities obtained from the growth chart, as well as fluctuations in weight, and signs of androgenization can allow the clinician to suspect cycling CD in prepubertal patients. Confirmation of cyclic CD diagnosis is only possible during periods of relapse (hypercortisolemic state) and should be investigated according to the current diagnostic standard.

## Background

Cushing's syndrome (CS) caused by an excess pituitary production of adrenocorticotropic hormone (ACTH) is referred to as Cushing' disease (CD), and is a rare disorder in prepubertal children. Moreover, CD which occurs cyclically is much less frequent, with unknown incidence in childhood. In children younger than 7 years, ACTH-independent causes of hypercortisolemia, primarily adrenal carcinoma, are more frequently seen than ACTH-dependent ones (1). According to recent data, after the age of 5 years ACTH-dependent CS is more common than ACTH-independent CS. In most cases, ACTH is produced by a pituitary microadenoma (2). The overproduction of ACTH occurs in a semi-autonomous manner and retains some auto-regulatory properties of the hypothalamic-pituitary-adrenal axis in the negative feedback cycle (3). In children, overproduction of ACTH causes hypercortisolemia resulting in obesity (BMI ≥ 1.5 SD) with simultaneous growth inhibition (growth velocity ≤ 0 SD), behavioral changes, and hyperandrogenization (hirsutism, precocious puberty without regression due to the suppression of gonadal axis by adrenal androgens). There are no symptoms of excess aldosterone (4). The prepubertal child with CD may present with obesity and growth failure alone, without other classical features such as plethora, hirsutism, acne, and striae (5).

Cyclic CS is characterized by repeated episodes of cortisol excess (relapses) interspersed by periods of normal cortisol secretion (remissions). The so-called cycles of hypercortisolism can occur regularly or irregularly with intercyclic phases ranging from days to years. Cycle lengths between 12 h and 85 days have been reported (6). Cyclic CS originates in 54% of cases from a pituitary corticotroph adenoma, in 26% from an ectopic ACTH-producing tumor and in about 11% from an adrenal tumor, the remainder being unclassified (7). To formally diagnose cyclic CS, three peaks and two troughs of cortisol production (clinical and/or biochemical) should be demonstrated, separated in time by intercyclic phases. The majority of patients with cyclic CS have clinical signs of CS, which can be either fluctuating or permanent. In a minority of patients, clinical signs of CS are absent. The fluctuating clinical picture and discrepant biochemical findings render cyclic CS extremely hard to diagnose. Clinicians should therefore be aware of this clinical entity and actively search for it in all patients with suspected CS but normal biochemistry or vice versa (7). Therefore, strict observation and repeated measurement of urinary cortisol are a reliable and convenient screening tool for suspected cyclic CS (8).

We present the case of a 7.8 year old girl whose symptoms of CS began in the pre-pubertal period and were cyclic. Her parents gave their written, informed consent for the presentation of the medical data and her photos in the journal article. Our national guidelines waived the requirement for ethical approval for the presentation of patients' medical data by the leading doctor.

## Case Report

**Figure 1:**
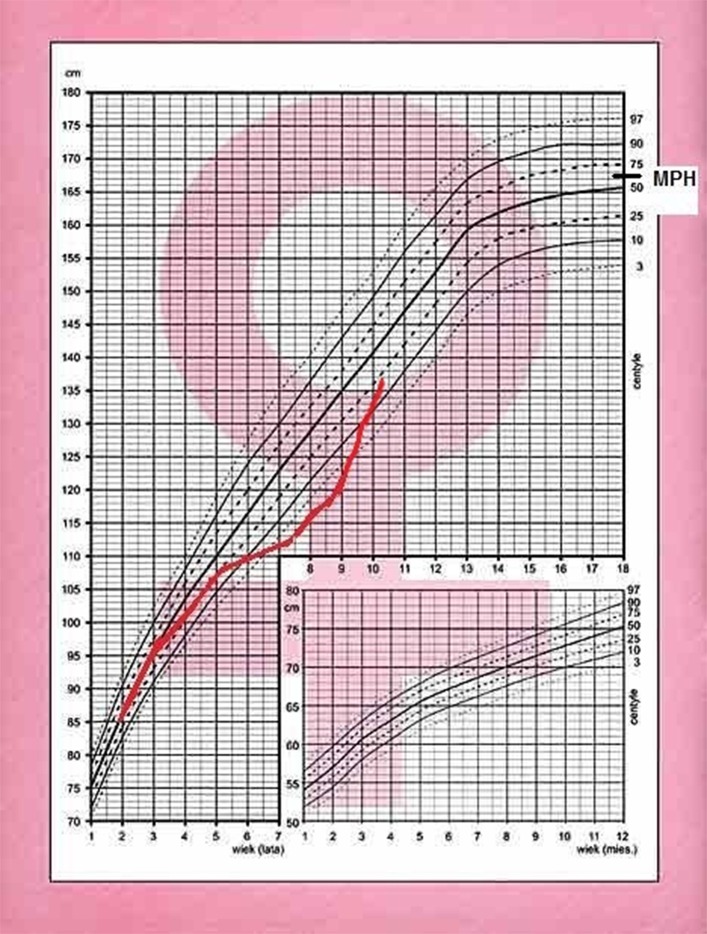
Growth chart of the patient with cyclic Cushing's disease.

A 7.8 year old Caucasian girl was brought by her mother to pediatric endocrinology due to short stature and excess of body mass. Upon the first visit the patient was in good clinical condition. Her height was 114 cm (below 3rd percentile) and her body weight was 24 kg (+14.7% excess of body weight appropriate for her height, and BMI 18.5 kg/m^2^, 87th percentile, *Z*-score 1.12). According to her mother, the patient had been obese 6 months prior but had lost 6 kg within the last 6 months due to dietary changes and lifestyle modification. The mother was also worried that the child was always the shortest in the peer group since kindergarten. The patient's growth curve was compiled from measurements reported in her medical records and showed that her growth was linear along the 3rd percentile during the past year, but a previous available measurement performed in the 2nd year of life was at the 25th percentile. Her mid-parental height (MPH) was between the 50th and 75th percentiles (Figure 1). Moreover, signs of precocious puberty were noticed: pubarche II stage according to Tanner scale without axillarche, and doubtful thelarche difficult to differentiate from steatomastia. Therefore, the girl was admitted to the ward for further diagnostics.

Sampling of urinary-free cortisol (UFC) in one 24 h urine collection, morning serum ACTH, cortisol, and 17-OHP concentrations were normal. GnRH stimulation test was performed and did not confirm central precocious puberty. Ultrasonography of the abdomen was normal. MRI of the hypothalamic-pituitary area was performed at the age of 8 and showed no deviations. However, because of a suspicion of potential cyclic hypercortisolemia [thanks to our previous experience with primary pigmented nodular adrenocortical disease (PPNAD) patients] the decision of a further follow-up observation was made.

**Figure 2:**
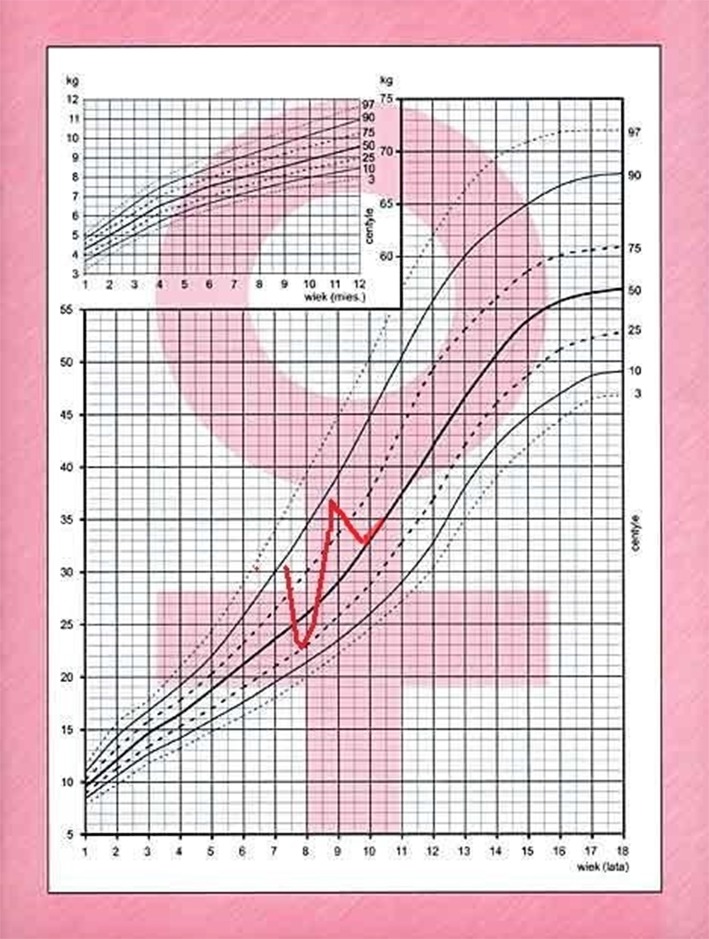
Body mass chart of the patient with cyclic Cushing's disease.

**Figure 3:**
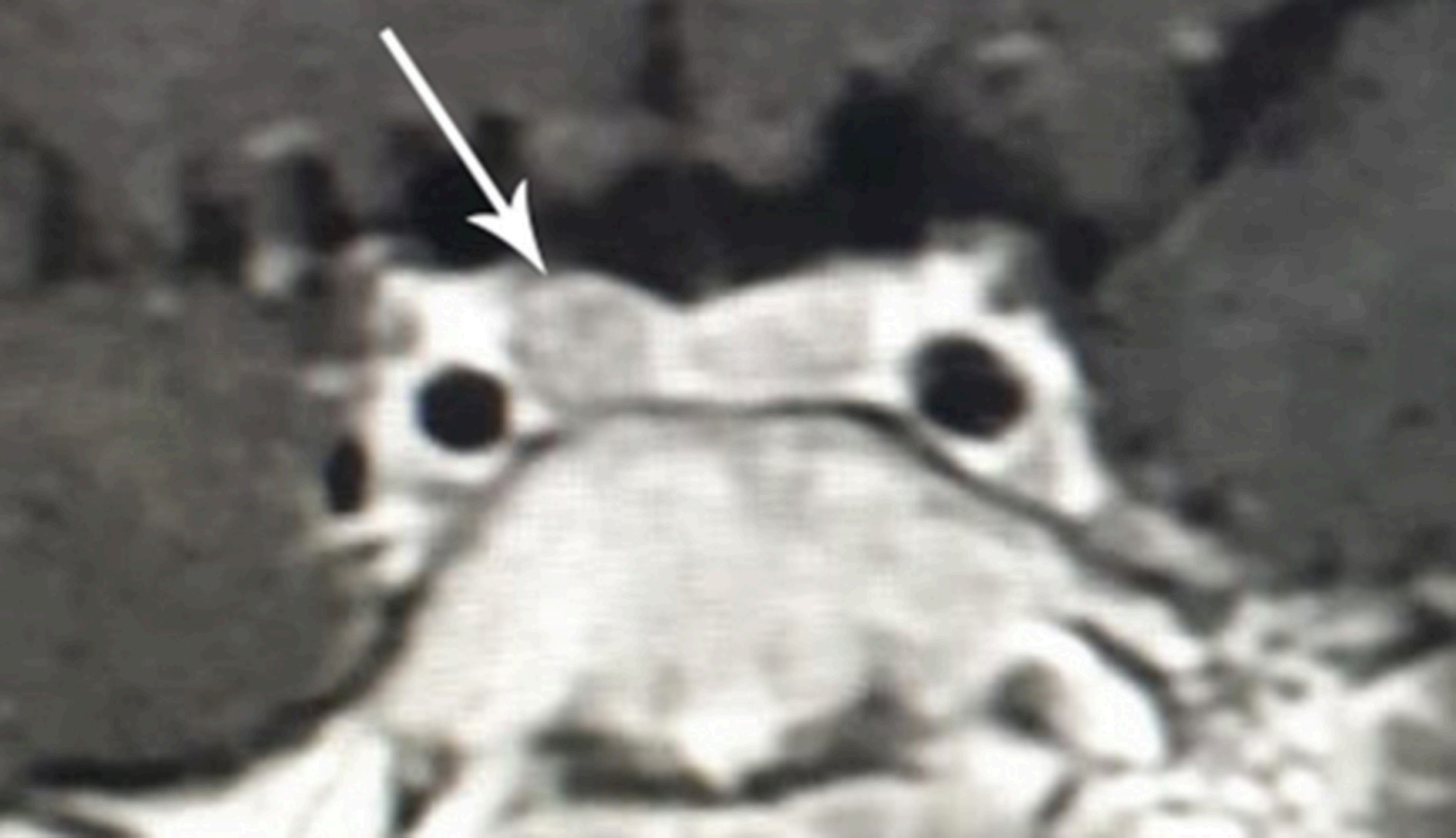
Pictures of the patient with cyclic Cushing's disease in symptomatic hypercortisolemia at the age 7 2/12 years, followed by remission at the age 7 8/12 years and in the relapse period at the age 8 6/12 years.

After 6 months, the patient returned with rapid weight gain and darkening of her skin, accompanied by nocturnal anxieties, exacerbation of depressive behavior, insomnia, and excessive sweating. She was therefore admitted to the ward for re-evaluation of the hypothalamo-pituitary-adrenal axis and to repeat the MRI of the hypothalamic-pituitary area. At 8.6 years, +57% excess body weight to height was visible, BMI was 23.6 kg/m^2^ (97 percentile, +5.8 SD, *Z*-score 2.46). Retrospective analysis of her growth chart showed inhibition of her growth velocity during last 3 years from 25 percentile to −2.4 SD, with rapid development of obesity (only during last year from +9% to +57%), varying in time (Figure 2). The photos of the girl presented by her mother revealed cyclic changes of her appearance in previous several months (Figure 3). At admission the girl was sad and depressive. Obesity was visible mainly on her face, neck, and abdomen, with areas of acanthosis nigricans on her neck (Figure 3), hirsutism on her back, thighs and above her upper lip. She presented with pubarche (II stage) in the absence of development of the breast glands (steatomastia). Blood pressure was normal. Bone age was in line with her chronological age of 8 years.

**Figure 4:**
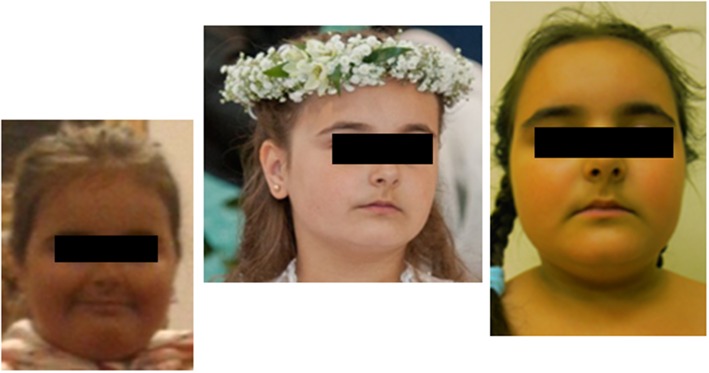
MR image of the hypothalamic-pituitary area with contrast with visible pituitary adenoma in the patient with cyclic Cushing's disease in the second relapse period (clinical and biochemical hypercortisolemia at the age of 8 6/12 years).

Because of the suspicion of CD, the diagnostic process was provided based on standard hormonal criteria: increased UFC in three 24 h urine collections, disturbances of cortisol circadian rhythm, increased or not suppressed plasma ACTH levels at 8.00 am accompanying increased serum cortisol levels, and a failure to suppress serum cortisol levels to <1.8 μg/dl during an overnight dexamethasone suppression test (ODST; 1 mg of dexamethasone was given at 11.00 pm). The pituitary etiology of CS was confirmed based on the serum cortisol suppression in a high-dose dexamethasone suppression test (2 mg q.i.d. for 48 h), a positive result of a corticotropin-releasing hormone (CRH) stimulation test (1 μg/kg, maximum 100 μg i.v.) and positive pituitary MRI. The above diagnostics have shown hypercortisolemia. Diurnal collection of UFC exceeded 10-fold the normal range [219, 284, and 283 μg/24 h respectively, normal range (*N*) <27 μg/24 h]. The temporal cortisolemic profile was reversed and without a night-time decline (8.00 a.m.−9.5 μg/dl, 8.00 p.m. −18.5 μg/dl, midnight −25.4 μg/dl). The ACTH concentration at the same time was 37.9 pg/ml (*N* 10–60 pg/ml), ruled in ACTH-dependent CS (no suppression (>20 pg/ml) with hypercortisolemia). Plasma renin activity (1.57 ng/ml/h, *N* 1.5 −5.7), aldosterone (85.4 pg/ml, *N* 35 −310), DHEA-S (36.5 μg/ml *N* <150) were within normal limits. After ODST, cortisol levels decreased (from 9.5 to 1.02 μg/dl) whereas the ACTH level was stable (34.2 pg/ml). The result of CRH stimulation test then confirmed CD with an increase in ACTH by 46% (>35%) and cortisol by 45% (>20%). MRI of the pituitary, repeated 6 months after the previous, which was normal, revealed an area of reduced signal that measured 3 × 4 mm located in the right-sided part of the anterior pituitary gland (Figure 4).

After pharmacological preparation (ketoconazole), the patient underwent an endoscopic transsphenoidal resection of the pituitary microadenoma. The histopathological examination revealed an atypical, densely-granulated pituitary corticotroph adenoma. The level of MIB1 antibodies, used to measure Ki-67 expression, was above 3%. Neurosurgery treatment resulted in the resolution of endogenous hypercortisolemia. The patient required hydrocortisone supplementation for 4 months after surgery. MRI of the pituitary gland performed 3 and 12 months after surgery, revealed postoperative changes, without tumor regrowth. Currently, one and a half years after surgery, her BMI is 21.7 kg/m^2^ (92th percentile, 1.42). The rate of her growth has improved (current height is between 10 and 25th percentiles). On exam, there are no symptoms of hypercortisolemia. All biochemical tests, assessing her hormonal axes were normal at 1 year.

Analysis of the clinical picture of the patient and the results of the tests performed in the hypercortisolemic (relapse) period indicated a diagnosis of cyclic CD (cyclic ACTH-dependent CS) induced by pituitary microadenoma, which was confirmed by histopathological examination, after a transsphenoidal tumor resection.

## Discussion

We present a case of cyclic CD in the prepubertal period. According to our knowledge, it is the first such description in medical literature. Both conditions, cyclic CD as well as CD, in prepubertal time are very rare in medical practice. Only several cases of cyclic CS in children have been described in prepubertal time. PPNAD and hyperplasia of adrenocoticotrophs were causes of cyclicity of CS in some reports (9, 10). However, one case of prepubertal cyclic CD was described (11), but the diagnosis is unclear. There was a boy who at the age of 4 started sudden, unexplained weight gain and for almost 8 years presented with several episodes of facial and abdominal swelling with concomitant hypertension, but without deterioration of his growth velocity, which occurred finally at the age of 12. At that time, in spite of repeated hypercortisolemia as displayed previously, ACTH level was periodically elevated. Computerized tomography suggested a filling defect in the pituitary and finally a basophil pituitary adenoma was removed. This patient at the age of 5.5 had a left atrial myxoma removed, and together with the repeated episodes of hypercortisolemia should suggest PPNAD in Carney complex (12). In the outcome of PPNAD, ACTH fluctuation could be also observed. There is no information about further outcome of the disease after neurosurgery in the described patient.

The most numerous group of cyclic CD was described by Alexandraki et al. (13). In the cited study, cyclicity was considered as the presence of at least one cycle, defined as a clinical and/or biochemical hypercortisolaemic peak followed by clinical and biochemical remission, followed by a new clinical and/or biochemical hypercotisolaemic peak. According to this definition our patient fulfills its criteria. Our patient could have been diagnosed with the cyclic form of the CD after just one cycle confirmed clinically and biochemically. The cycle lasted about 1 year. However, because of atypical signs and syndromes of CD in prepubertal time, we cannot be sure if more previous hypercortisolemia periods could have taken place before this one cycle described and confirmed clinically by the patient's mother, photos, and the analysis of patient's growth chart. Due to the insidious course of the disease and various long periods of remission and relapse, diagnosis usually lasts up to several years (6). The cyclical, insidious clinical course and atypical signs and symptoms of CD in prepubertal period may be responsible for the difficulties of its diagnosis. The knowledge of this phenomenon is important because, if not recognized, it can lead to failure in diagnosis of the disease. Significant cortisol fluctuations in CS were first recognized in 1956 by Birke et al. (14). However, the first description of cyclic CS is usually ascribed to Bailey who published another case in 1971 (15). He presented evidence for cycles of production in a patient with a slow-growing malignant lung tumor (carcinoid type). Since that time there were further sporadic and rare reports of this type of CS described. In the study of Alexandraki et al. who analyzed CD patients treated from 1946 to 2007 and described cyclicity in 30 (14.9%, 26 females) of them (13), the median number of cycles was two for each patient, and 4 years was the median intercyclic period. In multivariate analysis older patients, longer follow-up, female sex, and no histological identification of the adenoma were associated with an increased risk of cyclic disease. However, the study was retrospective and relied totally on patient recall of the clinical situation, and the reports by the physicians in the medical case records. In this long-time of observation different diagnostic methods were also used to diagnose CS. In this study only one of the 17 pediatric patients (5.88%) showed cyclicity, but detailed data about their age were not presented. Due to the periods of relapse and remission of the disease lasting variously for a long time (and thus diagnostic difficulties), the incidence of the disease is considered to be underestimated.

Atkinson's team also described some cases of cyclic CD diagnosed postoperatively, after transsphenoidal microsurgery for CD (16). The authors of this observation suggest that cyclic CD may be a much more common finding than previously recognized and emphasized the need for detailed and ongoing endocrinological investigation after pituitary surgery for hypercortisolism.

There is also an abstract presenting refractory cyclical CD diagnosed first time in a 3.8 year old girl in whom the whole anterior pituitary gland was removed and a relapse of CD occurred 6 years after surgery (17). The description of this case in our opinion indicate rather a relapse of non-cyclic CD following treatment, which has been reported in about 10–30% of patients with CD after neurosurgery (18, 19).

The pathophysiology of cyclic CS is largely unknown. One known cause of cyclic CS in childhood is PPNAD which is ACTH-non-dependent CS, moreover patients with PPNAD could present with other concomitant diseases (9, 12, 20). Several hypotheses have been suggested to explain cyclicity of CD, including episodic hemorrhage, the synchronous growth and death of tumor cells (7, 21) or fluctuations in adrenal-pituitary axis feedback (22). Nevertheless, the regularity of cyclic CD is difficult to explain by fluctuating cell growth and death only (7). Another theory supposes a hypothalamic origin for cyclic CD, with periodic changes in neurotransmitters contributing to pituitary ACTH secretion being involved. These neurotransmitters include CRH, noradrenaline, dopamine, acetylcholine, and gamma-aminobutyric acid. To summarize, ACTH secretion by a pituitary tumor could be regulated through autocrine or paracrine mechanisms, and lead to the long-term cycling seen in a fraction of CD cases.

In our case, the patient presented with CD symptoms in prepubertal time, and the diagnosis of cyclical CD was made at the age 8.5 years. There are no data about cyclic CD in childhood in the literature to compare with ours. The median age of CD diagnosis in children is 14.1 years old (23). The occurrence of such a rare disorder at such an early age may suggest a genetic basis of the disease. According to the latest literature, some mutations may play a role in the pathogenesis of Cushing's disease. In more than one-third of patients under the age of 18 years with CD, somatic ubiquitinase gene 8 (USP8) mutations were identified (24). The USP8 gene encodes a protein (with deubiquitinase activity) that inhibits lysosomal degradation of the epidermal growth factor receptor (EGFR), which in turn leads to increased expression of POMC and production of ACTH (25, 26). Another gene predisposing to corticotropinomas is CABLES1 (27). Mutations in this gene have been found in both children and adults with corticotropinoma. Genetic processes leading to CD are still not fully understood. Further research into the genetic background may allow us to identify the causes of CD and may be the basis for new therapeutic methods, especially in patients with cyclic outcomes of the disease. Therefore, the presented patient is an excellent candidate for potential genetic testing.

When cyclic CS is biochemically confirmed in childhood, further imaging, and laboratory studies are guided by the presence or absence of ACTH dependency. Suppressed ACTH levels suggest that the disease is located in the adrenal gland (PPNAD, hyperplasia, other tumors) and diagnostics should be directed toward these diseases (9). In cases of suspected ectopic ACTH production, specific biochemical testing for carcinoids or neuroendocrine tumors is required, including measurements of serotonin in platelets and/or urine, chromogranin A, and calcitonin. Differential diagnosis of cyclic CS should include following conditions: mild and subclinical CS, pseudo-Cushing's states, aberrant receptor mediates CS, factitious CS, and glucocorticoid resistance (7). All above-mentioned conditions were excluded in the presented case.

Although CD is the most common case of CS in childhood, in prepubertal age children, it is very rare and presents an important diagnostic and therapeutic challenge. Dias et al. presented 17 prepubertal children, with predominance of males (13M, 4F). All children had below normal linear growth and excessive weight gain at presentation. A high proportion (85% of males, 75% of females) had evidence of excessive virilization. Striae and hypertension were seen in 41% of patients (5). The difficulties associated with the clinical, biochemical, and radiological diagnosis of Cushing's disease in children are known and have been described before. Early diagnosis remains a challenge because test results often do not match standard diagnostic criteria (28, 29). The investigation of highest sensitivity (100%) for CD was excessive increase of serum cortisol to intravenous CRH (mean increase 113%) (5). Visualization of the pituitary microadenoma in MRI is not necessary for the diagnosis of CD, because tumors <3–4 mm in size are only found in about half of the cases. In two studies including pediatric patients, pituitary adenomas were found in 63 and 55% (18, 30). In our patient, the first MR image of the pituitary was normal but the next MRI (half a year later) showed a pituitary microadenoma. Although we did not perform bilateral inferior petrosal sinus sampling, this procedure can be more sensitive than pituitary imaging in localization of microadenomas and has been associated with improved cure rate by transsphenoidal selective adenomectomy (31).

## Conclusion

The cyclic clinical course and atypical signs and symptoms of CD in prepubertal period, may be responsible for the difficulties in diagnosis and its insidious outcome. The knowledge about this phenomenon is very important for the pediatrician to avoid long-term complications of failing to recognize Cushing's Disease and its consequences for the future health of the child.

## Data Availability Statement

All datasets for this study are included in the manuscript and the supplementary files.

## Ethics Statement

Written informed consent was obtained from the minor(s)' legal guardian/next of kin for the publication of any potentially identifiable images or data included in this article.

## Author Contributions

AW, AK-K, JS, BH, and GZ: medical and surgical practices. JS and AW: concept. AW, JS, and BH: design. BH, AW, and AK-K: data collection and processing. AW, JS, and AK-K: analysis and interpretation. AW and BH: literature search and writing.

### Conflict of Interest

The authors declare that the submitted work was carried out in the presence of any personal, professional or financial relationships that could potentially be construed as a conflict of interest.
